# Determinants of the adoption of climate smart agriculture practices by smallholder wheat farmers in northwestern Ethiopia

**DOI:** 10.1016/j.heliyon.2024.e34233

**Published:** 2024-07-06

**Authors:** Sindie Alemayehu, Zemen Ayalew, Million Sileshi, Fresenbet Zeleke

**Affiliations:** a School of Agricultural Economics and Agribusiness, Haramaya University, Haramaya, Ethiopia; b Department of Agricultural Economics, Bahir Dar University, Bahir Dar, Ethiopia; c Department of Agricultural Economics, Mekdela Amba University, Ethiopia

**Keywords:** Adoption, Climate smart agricultural practices, Multivariate probit, Ethiopia

## Abstract

Frequent climate variability and change had the strongest direct influences on the availability and accessibility of food through reducing agricultural productivity and cropping patterns. Despite the Ethiopian government having made substantial efforts to boost production and productivity through the introduction of Climate Smart Agriculture Practices (CSAPs), the implementation of these practices by smallholder wheat farmers has remained low. This study, therefore, tried to investigate the determinants of the adoption of CSAPs in Northwestern Ethiopia. The primary data were gathered from 385 randomly selected wheat producers (including 702 plot-level observations). The CSAPs considered in this investigation were wheat row planting, crop rotation, and improved wheat varieties. The factors that influence the adoption of CSAPs were determined using a multivariate probit (MVP) model. The results revealed that the age of the sampled wheat producer farmers, education level of sampled wheat farmers, livestock holding, contact with development agents, credit access, off-farm activities participation, distance to input supply institution, slope of the plot, and soil fertility status of the plot were the major determinants of the adoption of CSAPs. The study suggested that policy-makers and stakeholders should strengthen farmers’ skills by providing sufficient and effective short-term training. Moreover, encouraging mixed crop-livestock production systems, strengthening credit access, development agents, and access to near-input supply institutions are required to scale-up the adoption of CSAPs.

## 1 Introduction

### 1.1 Background of the study

Climate extremes and fluctuations in the climate pattern not only bring several risks to the agricultural sector but also have a significant impact on undernourishment, hunger, and food insecurity [1]. The small-scale rain-fed farming systems in Africa led the continent severely vulnerable to climate extremes and fluctuations [2]. The continent faces climate change related hazards, including more frequent extreme events like temperature fluctuation, erratic rainfall, floods, drought, and disease outbreaks [3]. These climate change hazards affect millions in sub-Saharan Africa by destabilizing agricultural productivity and cropping patterns, thus in turn impacting food quality, food availability, food accessibility, disrupting food prices, reliability of delivery, and overall welfare of rural households [4,5].

Ethiopia, located in Sub-Saharan Africa, is the country where the economy is dominated by the agriculture sector. Nonetheless, the bulk of Ethiopian farmers are smallholders who primarily engage in rain-fed farming, which is marked by low productivity and minimal application of agricultural technologies [6,7]. Since Ethiopian agriculture relies heavily on rain-fed farming methods, it is particularly susceptible to the impacts of climate change, which includes high temperature fluctuations, unpredictable rainfall, and recurrence droughts [8,9]. Ethiopia's agriculture sector is significantly impacted by climate change, which in turn affects the country's economy. For example, a study conducted by Gelaw [10] stated that by 2050, climate change is projected to decrease the gross domestic product (GDP) of Ethiopia by 8–10 %, but adaptation measures might reduce losses from climate shocks by half. Additionally, Solomon [11] predicted that by 2050, losses in agricultural GDP due to climate change will reach 31.1 %.

Wheat crop is a significant contributor to ensure food security and the overall economic growth of the agriculture sector in Ethiopia. It is a widely cultivated cereal crop that ranks third after *teff* and maize in terms of area coverage and second after maize in total volume of production [12]. Moreover, wheat is the second essential source of food energy and protein in Ethiopian nutrition, next to maize, which contributes 13.41 % of total calorie intake and 13.82 % of protein [13]. Similarly, East Gojjam stands out as the leading zone in terms of total area coverage, production, and productivity of wheat in the Amhara region [12]. In 2021/22, from 166,578 ha of land, 5.3 million quintals of wheat were produced. However, wheat production in the study area in particular and throughout Ethiopia in general is highly affected by climate change and variability and its production will decline by 25.5 % in 2050 [11]. The low level of productivity, which is exacerbated by climate change coupled with the increasing demand of wheat consumption in Ethiopia is expected to aggravate the existing food shortage in the country [14]. Thus, yield-enhancing technologies and practices should be widely adopted to achieve self-sufficiency in wheat production and ensure food security [15].

The Ethiopian government in collaboration with Food and Agricultural Organization (FAO) introduced CSAPs to enhance agricultural productivity sustainably; improve farmers' adaptation ability and resilience to climate change; and decrease and/or remove greenhouse gas (GHG) emissions in 2010 [16]. Climate Smart Agriculture Practices (CSAPs) could help to improve soil fertility, reduce erosion, break pests’ life cycle, reduce GHG emissions, suspend weeds, and hence improve crop productivity [17,18]. For instance, crop rotation is important to the reduce risk of pest and weed infestations, increase nitrogen fixation and formation of organic matter, improve water and nutrient distribution in the soil profile, and substantially lower GHG emissions [16]. Improved crop varieties and row planting reduce the risk of crop failure resulting from rainfall variability, thus enhancing productivity and resilience against climate change [19,20]. Different CSAPs like SWC practices, row planting, improved agricultural inputs, traditional small-scale irrigated farming, intercropping, and crop rotation are practiced to adapt to the effects of climate change in Ethiopia. However, particularly in the study region and throughout Ethiopia, adoption levels remain relatively low [9,16]. Hence, it is crucial to identify the factors that affect CSAPs adoption behavior of the farmers. This knowledge is vital for planners and policymakers to design effective policies and strategies that can stimulate the adoption of CSAPs, thereby increasing productivity and ultimately improving food security and overall livelihoods without harming the environment. The investigation will also essential as source of information for future researches by identifying the adoption determinant of CSAPs.

### 1.2 Statement of the problem

Climate change is a major source of risk for smallholder farmers, which is related to low agricultural productivity, food insecurity, malnutrition, and poverty [21]. Ethiopia is often cited as an example where food insecurity prevails in its most distressful condition and is the largest recipients of food aid in Africa [22,23]. The food security situation in Ethiopia heavily depends on the performance of the agriculture sector. However, the nation's agriculture sector is heavily susceptible to climate change, specifically erratic and unreliable rainfall, temperature fluctuation, inefficient use of agricultural resources, land degradation, and poor agricultural intensification [24,25].

The prevalence of food insecurity and low agricultural productivity is a major development challenges in the east Gojjam zone of the Amhara region. According to Ref. [26], approximately 65.3 % of the households in the zone are food insecure, leading to a decrease in both the quality and quantity of diet to manage the stress of food insecurity. This situation is exacerbated by various factors, including soil degradation, disease, insect pests, decreasing farm size, the use of local varieties, unpredictable rainfall, and periodic droughts due to climate change [27]. For instance, the productivity of wheat is lower than its potential yield due to water stress (drought), disease, and insect pests [28,29]. Data from the Central Statistical Agency [12] indicates that the average productivity of wheat in the east Gojjam zone is 31.84 qt/ha, which is higher than the average productivity of Ethiopia (31.11 qt/ha) but lower as compared to the world average productivity (35.47 qt/ha) and also other African countries average productivity such as Egypt (64.5 qt/ha) and South Africa (43.12 qt/ha) in 2021 [30].

Climate smart agriculture practices (CSAPs) are vital to sustainably fostering agriculture and improving food security [31,32]. The government of Ethiopia has introduced various policies and strategies related to the adoption of CSAPs to boost agricultural productivity and strengthen resilience against climate change. For instance, Ethiopia's National Adaptation Plan (NAP-ETH) builds on existing initiatives to mitigate climate vulnerability by developing adaptive capabilities and resiliencies. One of the pillars of NAP-ETH is improving food security through CSAPs, thereby ensuring sustainable growth in agricultural production [33].

Following this, the determinants of adoption of CSAPs in Ethiopia have been examined by a few studies. Some studies focused on other crops than wheat [[34], [35], [36]], while others focused on factors affecting the adoption of a single CSAP. For instance Refs. [[37], [38], [39]], examined the adoption of wheat row planting, and [40,41] examined the adoption of improved wheat variety. However, these studies have been limited in terms of identifying how practices complement each other, despite wheat farmers employing various CSAPs simultaneously to adapt and mitigate to climate change. Moreover, some of the studies on the determinants of adoption of CSAPs modeled as binary and multinomial [42,43]. These studies didn't consider the possible interdependency between different CSAPs. In fact, adoptions of CSAPs are interdependent household decisions that should be estimated simultaneously. Besides, most of the former studies were conducted at the household level and did not consider plot-specific characteristics. Hence, in contrast to prior studies, this study considers interdependent and simultaneous adoption decisions using a multivariate probit model, that considers the expected simultaneity problem in the households' adoption decisions and interdependence between different CSAPs [44]. Furthermore, this paper is based on plot-level analysis, which includes plot-specific characteristics in the analysis.

### 1.3 Objective of the study

This paper aimed to investigate the determinants of the adoption of CSAPs, including wheat row planting, crop rotation, and improved wheat varieties in Northwestern Ethiopia.

## 2 Conceptual and theoretical framework

The theory of adoption of agricultural technology is a broad idea that helps to understand why certain farmers adopt new technologies while other farmers do not adopt it. The body of literature on this topic is based on three principal paradigms: the adopter-perception paradigm; the diffusion-innovation paradigm; and the economic constraint paradigm [45,46]. The adopter-perception paradigm posits that the adoption process begins when farmers perceive a need for innovation. This perception is influenced by personal factors (like human value, education, and experience) and physical characteristics of the land [46]. Farmers' preference for specific technological traits can significantly influence their adoption behavior. Technology adoption by farmers often involves rational decision-making, shaped by their perceptions of the technology's suitability and its delivery, along with cultural, contextual, and individual factors [45,46]. Unlike the economic constraint paradigm, which focuses strictly on financial factors, this paradigm broadens the definition of utility to include a wider range of considerations [47].

The innovation-diffusion paradigm explains the adoption of agricultural technologies over time through communication, information, and knowledge [46]. The innovation-diffusion paradigm assumes that access to information is crucial in influencing adoption decisions [48]. According to this theory, adopting an innovation involves a cognitive process consisting of five stages: technology awareness, persuasion, decision-making, implementation, and confirmation [49,50]. In contrast, the economic constraint paradigm posits that farmers contend to maximize profit or utility, with disparities in resource distribution being key factors influencing adoption behavior [45]. This paradigm emphasizes the significant role of economic factors at the individual level in figuring out adoption decisions [47].

**Fig. 1:**
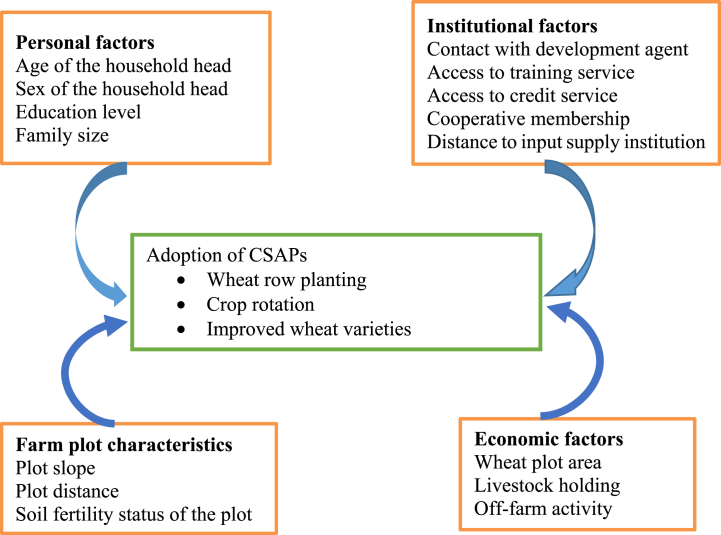
Conceptual framework of adoption of CSAPs.

Drawing from the theories and assumptions discussed earlier, the conceptual framework of this study is structured to explore the factors influencing the adoption of CSAPs through various paradigms (see in Fig. 1). Previous research has often employed econometric models integrating variables from these paradigms [45,50,51]. Accordingly, in this study, the adopter-perception theory is characterized using socio-demographic (personal) factor variables and plot-specific characteristics. The innovation-diffusion theory has been characterized through the institutional factor variables, specifically, contact with the development agent, access to training, credit access, membership in cooperatives, and distance to the input supply institution. Adesina and Zinnah [45] stated that engaging extension services, media, and local opinion leaders, experiment stations visits, and on-farm trials can persuade hesitant farmers to adopt new practices, emphasizing rational decision-making. Besides, economic variables like wheat plot area, livestock holding, and participation in off-farm activity capture aspects of the economic constraint theory.

## 3 Research methodology

### 3.1 Description of the study area

**Fig. 2:**
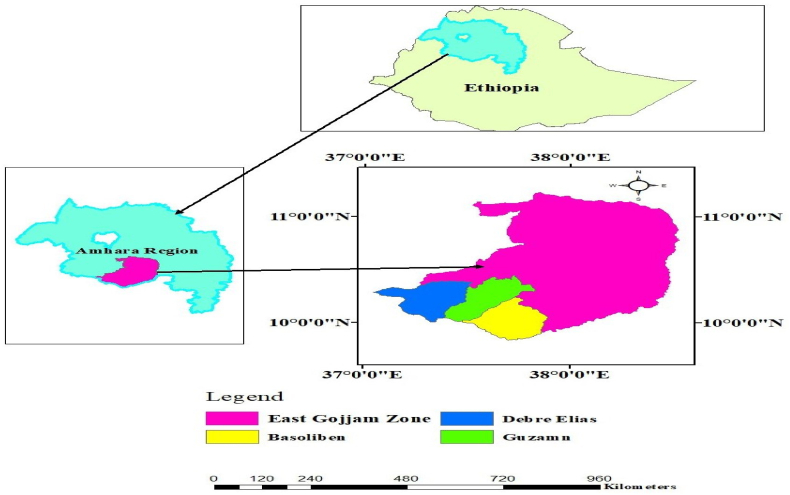
Map of the study area.

The study area, East Gojjam zone is an administrative region within the Amhara National Regional State (ANRS), which is located in the northwestern part of Ethiopia (see Fig. 2). The zone encompasses sixteen districts and four urban administrations, covering a total land area of 14,010 km^2^. Climate of east Gojjam varies, with an average annual rainfall ranging from 900 to 1800 mm. The region experiences a short rainy season (Belg) typically occurring in February and March, particularly in highland agro-ecology. The average temperature ranges from 7.5° C - 27° C throughout the year. Mixed farming system; crop production and livestock rearing is the main economic activity practiced in the east Gojjam zone. It is recognized as a significant agriculture area within the Amhara National Regional State, often supplying surplus crops for urban market. Among the crops produced in the zone, wheat stands out the first crop, contributing significantly to the total food grain production, followed by maize and *teff*, which accounts for nearly 29 %, 24 %, and 23 % of the total food grain production, respectively [27]. The production and productivity of wheat in Ethiopia and the east Gojjam zone are presented in Table 1.

**Table 1:** Wheat crop production and productivity in Ethiopia and east Gojjam zone.

	Ethiopia			East Gojjam		
Year	2019/20	2020/21	2021/22	2019/20	2020/21	2021/22
Area (hr)	1,789,372.23	1,897,405.05	1,867,047.71	127,350.06	147,694.69	166,577.99
Production (qt)	53,152,703.28	57,801,305.96	58,078,220.52	3,976,853.24	4,743,813.86	5,303,301.67
Productivity (qt/hr	29.70	30.46	31.11	31.23	32.12	31.84

However, agriculture in the region is predominantly reliant on rain-fed farming systems, rendering the sector highly susceptible to the effects of climate change and variability. The prevalence of diseases and insect pests, soil degradation, unreliable rainfall, temperature rise and variability, and recurrent drought are the major climate change-related problems that compromise the sustainability and productivity of agriculture [27]. In response to these, different CSAPs have been adopted to a limited extent. The scope of CSAPs in northwestern Ethiopia is broad and encompasses various strategies to enhancing productivity, resilience, and improving food security in the area of climate change. One area is sustainable land and soil management, which includes soil and water conservation measures (terracing, contour farming, digging ditches, and grass cover), as well as soil fertility management through the use of inorganic and organic fertilizers and green manure. Water resource management is another critical component, with efforts focused on expanding and improving irrigation systems and constructing rainwater harvesting structures to capture and store water for agricultural use.

Crop and livestock diversification is encouraged by promoting drought-resistant crop varieties and integrated crop-livestock systems to optimize resource use and mitigate risks. Improved agricultural practices such as conservation agriculture, which includes minimum tillage, row planting, crop rotation, and cover cropping are implemented to enhance soil health and water retention. Besides, agroforestry and tree planting are promoted to integrate trees farmlands, thereby improving soil fertility, providing shade, and producing fruits and timber, alongside efforts to restore degraded forests. The key initiatives and programs supporting these efforts in the study region include the Sustainable Land Management Program (SLMP), which focuses on land rehabilitation and sustainable agriculture practices; and the Productive Safety Net Program (PSNP), which integrates CSAP components to build community resilience. Fig. 2 presents map of the study area.

### 3.2 Description of variables and hypothesis

In this study, the adoption of CSAPs is considered as the dependent variable. Different CSAPs were adopted by smallholder wheat farmers in the study region. The most commonly implemented CSAPs were wheat row planting, crop rotation, and improved wheat varieties. Smallholder farmers used these CSAPs either solely or simultaneously on their plots. Key informant interviews (KII) have highlighted that wheat row planting is a crucial agricultural practice that holds significant importance in enhancing crop productivity. By arranging wheat seeds in rows and effectively distributing fertilizers, this method promotes optimal growth conditions for the crop, and facilitates efficient weed management, as it allows for targeted weed control measures to be implemented. The data obtained from FGD and KII showed that crop rotation is effective in lessening the incidence of pests, and suppressing weeds dispersion. Improving wheat varieties is essential to reduce crop failure risks caused by low and uneven rainfall patterns in different agro-ecological zones. The detail description of the dependent variables (adoption of CSAPs) and independent variables hypothesized to affect the adoption of these practices is presented in Table 2. Independent variables included in the model were selected through reviewing theoretical and empirical literature.

**Table 2:** Variables definitions, measurements, and hypothesis used in the MVP model.

Variables	Measurement	Hypothesis
**Dependent variables**		
Adoption of wheat row planting	Dummy (1 = adopter, 0 = non-adopter)	
Adoption of crop rotation	Dummy (1 = adopter, 0 = non-adopter)	
Adoption of improved wheat varieties	Dummy (1 = adopter, 0 = non-adopter)	
**Independent variables**		
Sex	Dummy (male = 1, female = 0)	+
Age	Year	+/−
Education level	Year of schooling (grade)	+
Family size	Adult equivalence	+
Wheat plot area	Hectare	+
Total livestock holding	Tropical livestock unit	+
Access to training	Dummy (1 = yes, 0 = no)	+
Access to credit	Dummy (1 = yes, 0 = no)	+
Contact with development agent	Number of days	+
Off-farm activities	Dummy (1 = yes, 0 = no)	+
Distance to input to supply institution	Walking minutes	–
Membership to cooperatives	Dummy (1 = yes, 0 = no)	+
Slope of the plot	Dummy (1 = flat slope, 0 = steep slope)	–
Plot distance	Walking minutes	–
soil fertility status of the plot	1 = Fertile, 2 = medium fertile, 3 = less fertile	+

### 3.3 Sample size and sampling technique

The sampled wheat farmers were selected using purposive and two-stage random sampling procedures. East Gojjam zone was chosen purposively due to the leading zone of wheat production in the Amhara region [12]. Additionally, the zone is vulnerable to problems induced by climate change, such as land degradation, recurrent drought, parasitic weeds, disease, and insect pests [27]. Three districts (Debre Elias, Gozamin, and Basoliben) in the east Gojjam zone were chosen purposively due to the presence of high wheat production areas and vulnerable to problems induced by climate change [52,53]. Then, in the first stage, seven *kebeles* were randomly selected in proportion to the number of *Kebeles* in each district. In the second stage, wheat producer households were identified within these selected rural *Kebeles*, and then a total of 385 wheat producer farmers (702 plots cultivated in the 2021/2022 production period) were randomly chosen using a probability proportional sampling method based on the numbers of wheat producer farmers in each *Kebele* (Table 3).

**Table 3:** Selected districts, kebeles, and their sample sizes.

Sampled districts	Kebeles	Total hhs	Sample hhs
Gozamen	Enerata	1168	50
	Wonka	1142	49
	Libanos	1372	59
Debre Elias	Gofichima	1636	49
	Yemezegn	1300	39
Basoliben	Dendegeb	2019	78
	Limchi	1558	61
Total		10,195	385

The total sample size was determined using a formula designed to ensure the desired level of precision (acceptable error), as outlined by Kothari [54]. This method is considered essentially appropriate in a situation with a large or unknown population divided into distinct subgroups or strata. According to Ref. [55], assume that when the population is large and the variability of the proportion “p” is not known, assuming p = 0.5 with a 95 % confidence level and ±5 % precision will yield sufficiently large sample size to ensure accurate predictions.

$$n=\frac{Z^{2}\;pq\;}{e^{2}}=385$$

where, n refers sample size; Z refers the standard cumulative distribution that corresponds to the level of confidence with the value of 1.96; e refers the level of precision its value equals to 0.05; p refers the estimated proportion of an attribute present in the population, with a value of 0.5 as recommended by Ref. [55] to get the desired minimum sample size of households at a 95 % confidence level and ±5 % precision; q = 1-p.

### 3.4 Data collection method

This study gathered qualitative and quantitative data from primary and secondary sources by trained enumerators and supervisors. Primary data were collected through face-to-face personal interviews using a semi-structured questionnaire. The questionnaire was pre-tested by 15 randomly selected wheat farmers, 5 from each district, before conducting the interviews for appropriateness and necessary modifications. The type of primary data collected from the sampled wheat farmers includes demographic characteristics of the household (age, sex, family size, and education), economic factors (livestock holding, number of wheat plots, wheat plot area, and off-farm activities), social and institutional factors (contact with development agent, access to training, distance from input supply institution, cooperative membership, and access to credit), and wheat plot characteristics (soil fertility status of the plot, slope of the plot, and plot distance). Additionally, the type of CSAPs implemented in wheat farmers’ plots were collected. Structured interviews were augmented by focused group discussions (FGDs) and key informant interviews (KIIs) with different individuals at different levels. The FGDs and KIIs, held using a checklist, helped to identify major CSAPs practiced in the area and also used to guide the design of household questionnaires.

Secondary data were collected on various aspects, including population statistics, the type of CSAPs implemented in the area and their scope, wheat production, and productivity figures, the list of wheat producer districts and *Kebeles*, and information about climate change and its impact to supplement primary data. The sources of information for secondary data include the Central Statistical Agency (CSA) and published and unpublished documents from zone and district offices of agriculture and rural development.

### 3.5 Method of data analysis

Farmers often use a combination of CSAPs to tackle multiple challenges depending on the constraints and benefits of the CSAP. One practice adoption decision may be conditioned by the choice of other practices due to their interdependence, either as complements or substitutes. Employing a univariate logit or probit model may lead to biased conclusions because they fail to capture the potential correlations of unobserved disturbances between different adoption decision equations [56].

Accordingly, the study employed the MVP model to identify the determinants of smallholder farmers’ adoption of CSAPs simultaneously. Following [57], the MVP econometric approach used in this research is characterized by a set of m binary dependent variables Y_im_, in the following equations:

(1) $$Y_{\text{im}}^{*}=\beta_{m}X_{\text{im}}+\varepsilon_{\text{im}},m=1\ldots m\;\left(\text{adoption}\;\text{of}\;\text{CSAPs}\;\text{choices}\right)$$

(2) $$Y_{im}=\left\{ \begin{array}{c} {1\;if\;Y}_{\text{im}}^{*}>0\\ 0{\;if\;Y}_{\text{im}}^{*}<0 \end{array}\right.\;and$$

where, $Y_{\text{im}}^{*}$ in Eq (1) refers a latent variable; $Y_{im}$ in Eq (2) refers the dependent variable; $X_{\text{im}}$ in Eq (1) refers a vector of explanatory variables; ***β***m are the unknown parameters; and $\varepsilon_{\text{im}}$ are random error components. The random error terms are distributed as a multivariate normal distribution with zero mean and unitary variance. The covariance matrix is presented in Eq (3):

(3) $$⟦\begin{array}{c} \varepsilon_{1i}\\ \varepsilon_{2i}\\ \varepsilon_{mi} \end{array}⟧\approx N\left[\left(\begin{array}{c} 0\\ 0\\ 0 \end{array}\right)\left(\begin{array}{ccc} 1 & \rho_{12}\; & \rho_{13}\\ \rho_{21} & 1 & \rho_{23}\\ \;\rho_{31\;}\; & \rho_{32} & 1 \end{array}\right)\right]$$

The off-diagonal elements in the covariance matrix, as outlined in Eq (3), represents the correlation (unobserved) among the error terms of multiple CSA adoption practices. If the off-diagonal elements are non-zero, it allows for covariance across the error terms and validates that the use of the MVP model instead of a univariate probit model. In this study, the value of off-diagonal elements was non-zero, implying that the dependent variables are interdependent. Thus, the MVP model was the appropriate econometric model to estimate the parameters.

#### 3.5.1 Diagnostic tests

Before fitting into the MVP model, explanatory variables were tested for multicollinearity problems using the Variance Inflation Factor (VIF) for continuous explanatory variables and the Contingency Coefficient (CC) for discrete choice variables. According to Ref. [58], variables with a VIF value greater than 10 were deemed to be highly collinear. Discrete choice variables with a CC value greater than 0.75 indicate the presence of collinearity in the data [59]. The test result indicates that the mean VIF value is 1.126, which is below 10 for all the variables. Similarly, the CC value for all discrete choice variables is less than 0.75 (see Appendix Table 1, Table 2). These indicate that there are no multicollinearity issues in the dataset. Breusch-Pagan/Cook-Weisberg test was used to test the existence of heteroscedasticity. The test result shows that (Chi^2^ = 34.80; Prob = 0.000) there exist heteroscedasticity problem in the model. Thus, we use robust standard error to correct heteroscedasticity. Besides, the Ramsey RESET test was employed to test the existence of missing variables. The Ramsey RESET test statistic [F (3, 682) = 0.39; Prob = 0.7610] illustrated that the model had no omitted variables.

## 4 Result and discussion

### 4.1 Climate smart agriculture practices adoption status of the household

The type of CSAPs implemented in 702 plots of smallholder wheat farmers in the study region are illustrate in Table 4. Based on the data gathered from FGD participant smallholder farmers, frequent growing of cereals on the same land year after year significantly reduces crop productivity. However, farmers continuously cropping cereals year after year on the same plot. In the study area, farmers cultivate wheat following *nug* or *teff* or maize, *teff* following wheat or maize or barley, maize next to wheat or *teff* or barley, barley after *teff* or maize, and *nug* after *teff.* Thus, the conventional crop rotation practice (planting cereals after cereals) was most prevalent in the study region. The rotation of cereals with legumes is not common in the area because cropping of food legumes was greatly decreased as a result of pests, disease, and acidity of the soil. The information obtained from KII shows that kekeba and ogoloncho wheat varieties were mostly adopted by wheat producer farmers in the study region. Likewise, the FGD information demonstrated that farmers most widely prefer the kekeba wheat variety due to its high productivity, disease resistance capacity, and earliness to escape terminal moisture stress. Similarly, the ogoloncho wheat variety had good adaptation, high yield, and disease resistance character which was preferred by the farmer. However, during the short rainy season, late maturity and low productivity were the limitations of the ogoloncho wheat variety.

**Table 4:** CSAPs adoption status of the plot of sampled households.

CSA practices	Wheat row planting		Crop rotation		Improved wheat variety	
	Freq.	Percent	Freq.	Percent	Freq.	Percent
non-adopter	153	21.79	386	54.99	360	51.28
Adopter	549	78.21	316	45.01	342	48.72
Total	702	100.00	702	100.00	702	100.00

### 4.2 Demographic and socio-economic characteristics of the sampled wheat farmers

The result in Table 5 showed that among eight continuous explanatory variables, six variables exhibited a mean difference that was statistically significant among adopters and non-adopters of CSAPs.

**Table 5:** Descriptive statistics for continuous variables.

Variables	Row planting		Crop rotation		Improved wheat variety	
	Adopter	Non-adopter	Adopter	Non-adopter	Adopter	Non-adopter
Age of the hh	44.47 (11.35)	46.73** (12.44)	44.75 (11.15)	45.13 (12.01)	45.09 (12.08)	44.83 (11.19)
Education	2.87 (3.28)	2.68 (2.99)	3.12 (3.33)	2.59** (3.11)	3.23 (3.42)	2.45*** (2.98)
Family size	4.91 (1.69)	4.79 (1.79)	4.95 (1.68)	4.83 (1.74)	4.89 (1.64)	4.88 (1.78)
Livestock	8.6 (4.46)	7.8** (3.52)	8.28 (4.26)	8.55 (4.31)	8.93 (4.43)	7.94*** (4.09)
Plot area	0.53 (0.25)	0.57* (0.27)	0.54 (0.25)	0.53 (0.26)	0.54 (0.26)	0.53 (0.25)
No of DA contact	18.25 (11.37)	13.63*** (8.56)	18.41 (11.93)	16.29** (10.06)	18.76 (11.82)	15.81*** (9.94)
Plot distance	25.07 (15.05)	26.76 (16.49)	24.87 (15.52)	25.91 (15.26)	24.77 (14.73)	26.09 (15.96)
Input distance	30.48 (17.91)	31.60 (23.24)	28.15 (16.89)	32.84*** (20.66)	28.95 (17.35)	32.42** (20.67)

Age of the sampled household head, contact with the development agents, livestock holding, and wheat plot area had significant mean differences among adopters and non-adopters of wheat row planting practices. The mean test analysis showed that there is a significant mean difference among adopters and non-adopters of crop rotation in terms of mean education level of the sampled household heads, contact with the development agents, and input supply institution at the 5 %, 5 %, and 1 % significant levels, respectively. Similarly, the analysis of the data demonstrated that there is a significant difference between the mean education level of the sampled household heads, livestock holding, contact with the development agents, and distance to input supply institution between user and non-user of improved wheat varieties.

The association between each discrete predictor variable and the adoption of CSAPs was conducted by cross-tabulating and *Chi*^*2*^ tests. Table 6 shows that the slope of the plot, credit access, participation in off-farm activity, and training access had a significant relationship with the implementation of wheat row planting practice. Crop rotation was significantly related with the participation in off-farm activity. Participation in off-farm activity and credit access had significant associations with the use of improved wheat varieties.

**Table 6:** Descriptive statistics for discrete choice variables.

Variable description		Row planting			Crop rotation			Improved seed		
		Adop ter (%)	Non-adop (%)	Χ^2^-value	Adopter (%)	Non-adop (%)	Χ^2^-value	Adop ter (%)	Non-adop (%)	Χ^2^-value
Sex of the hh	Male	89.62	86.27	1.35	88.92	88.86	0.001	89.77	88.06	0.52
	Female	10.38	13.73		11.08	11.14		10.23	11.94	
Plot slope	Flat	70.31	60.13	5.71**	67.09	68.91	0.27	67.54	68.61	0.09
	Steep	29.69	39.87		32.91	31.09		32.46	31.39	
Soil fertility status of the plot	Fertile	6.92	9.8	1.59	5.38	9.33	4.16	8.77	6.39	2.78
	Medium	65.76	62.09		65.51	64.51		66.08	63.89	
	Less	27.32	28.10		29.11	26.17		25.15	29.72	
Credit access	Yes	40.44	26.14	10.45***	39.87	35.23	1.60	41.52	33.33	5.03**
	No	59.56	73.86		60.13	64.77		58.48	66.67	
Training access	Yes	47.91	38.56	4.21**	44.30	47.15	0.57	48.25	43.61	1.52
	No	52.09	61.44		55.70	52.85		51.75	56.39	
Cooperative membership	Yes	81.06	75.16	2.58	81.01	78.76	0.55	80.70	78.89	0.36
	No	18.94	24.84		18.99	21.24		19.30	21.11	
Off-farm activity	Yes	29.69	17.65	8.79***	22.78	30.57	5.33**	23.68	30.28	3.86**
	No	70.31	82.35		77.22	69.43		76.32	69.72	

### 4.3 Determinants of the adoption of climate smart agricultural practices

The maximum likelihood estimation results of the MVP model to show the determinants of the adoption of different CSAPs among smallholder farmers' plots is presented in Table 7. The Wald chi^2^ test is 145.80, statistically significant at a 1 % significant level. This result revealed that the MVP model adequately fits the data, leading to the rejection of the null hypothesis that all the coefficients in each equation being equal to zero. The null hypothesis that there is no correlation among the three equations error terms (rho21 = rho31 = rho32 = 0: chi^2^ (3) = 35.4922 Prob = 0.0000) is strongly rejected at a 1 % significant level. This result confirms that farmers’ adoption decisions between the three CSAPs are interdependent decisions. Thus, the separate estimations of the adoption decisions of the three CSAPs are biased. Hence, the MVP model is justified to a greater extent compared to the univariate probit model. The correlation coefficients between wheat row planting and crop rotation, between wheat row planting and improved wheat varieties, and between crop rotation and improved wheat varieties were 33.2 %, 18 %, and 17.9 %, respectively. A positive sign indicates that the interactions among CSAPs were complementary, meaning that they were used simultaneously by the respondents.

**Table 7:** Determinants of adoption of CSAPs: MVP method.

Variables	Row planting			Crop rotation			Improved wheat varieties		
	Coef.	Std. Err.	Z	Coef.	Std. Err	Z	Coef.	Std. Err	Z
**Demographic and economic variables**									
Sex	−0.128	0.176	−0.730	−0.084	0.165	−0.510	0.026	0.164	0.160
Age	−0.010*	0.005	−1.860	−0.001	0.005	−0.120	0.004	0.005	0.840
Education	−0.005	0.017	−0.290	0.039**	0.017	2.390	0.050***	0.017	3.030
Family size	0.040	0.036	1.110	0.044	0.032	1.400	0.000	0.032	0.000
Livestock	0.036***	0.013	2.810	−0.012	0.012	−1.000	0.029**	0.012	2.410
Off-farm	−0.361***	0.131	−2.760	0.300***	0.112	2.680	0.262**	0.112	2.350
**Plot specific characteristics variables**									
Plot area	−0.247	0.214	−1.150	0.126	0.195	0.640	0.180	0.195	0.920
Plot slope	−0.352***	0.136	−2.590	−0.025	0.123	−0.210	0.142	0.124	1.140
Plot distance	−0.003	0.004	−0.750	−0.002	0.003	−0.620	−0.002	0.003	−0.450
Soil fertility status (fertile soil as reference)									
_Medium fer	0.269	0.205	1.310	0.357*	0.191	1.870	−0.159	0.190	−0.840
_Less fertile	0.425*	0.236	1.800	0.487**	0.219	2.230	−0.279	0.219	−1.270
**Institutional variables**									
DA contact	0.021***	0.006	3.280	0.010**	0.005	2.130	0.013***	0.005	2.770
Credit access	0.243**	0.121	2.020	0.096	0.104	0.930	0.178*	0.103	1.730
Coopmember	0.137	0.143	0.960	0.035	0.129	0.270	0.017	0.123	0.140
Training	0.109	0.110	0.990	−0.090	0.098	−0.910	0.101	0.099	1.020
Input distance	−0.002	0.003	−0.550	−0.008***	0.003	−3.140	−0.005*	0.003	−1.840
cons	0.661	0.430	1.540	−0.781**	0.391	−2.000	−0.959**	0.404	−2.370
/atrho21	0.345***	0.070	4.890		Multivariate probit (MSL, # draws = 100)				
/atrho31	0.182***	0.068	2.690		Log pseudolikelihood = −1246.5512				
/atrho32	0.181***	0.062	2.940		Wald chi2 (72) = 145.80				
rho21	0.332***	0.063	5.290		Prob > chi2 = 0.0000				
rho31	0.180***	0.065	2.750		Number of obs = 702				
rho32	0.179***	0.060	3.010		Likelihood ratio test of rho21 = rho31 = rho32 = 0: chi2 (3) = 35.4922 Prob > chi2 = 0.0000				

Note: ***, **, and * refers to significance at 1, 5, and 10 % significant levels, respectively.

The MVP model regression result in Table 7 demonstrated that the adoption decisions of the three CSAPs were affected by different variables. The household's decision to adopt wheat row planting was significantly determined by the age of the sampled household heads, livestock holding, contact with development agents, participation in off-farm activity, credit access, plot slope, and soil fertility status of the plot. Crop rotation was determined by the education level of the sampled household heads, contact with development agents, participation in off-farm activity, soil fertility status of the plot, and distance to input supply institutions. Improved wheat variety was determined by the education level of the sampled household heads, livestock holding, contact with development agents, participation in off-farm activity, credit access, and distance to input supply institutions.

The age of the sampled household head had a significant negative association with the adoption of wheat row planting at a 10 % significant level. This might be due to the risk aversion behavior of farmers, which is expected to rise as the age of the household head increases. Aged farmers are frightened to adopt new technologies and practices that involve significant risks with higher returns tradeoffs. A study conducted by Ref. [42] found that the likelihood of adopting land management practices decreases as the age of the household head increases. Younger household heads having longer planning horizons, being more flexible, and greater risk-taking behavior are more prone to adopt new ideas and novelties. Similarly, Haq et al. [60] found that the adoption of CSAPs in Pakistan was negatively affected by age. In contrast, an investigation carried out in Vietnam discovered that aged farmers have accumulated experience and knowledge over time from adapting to shocks associated with climate changes. As a result, farmers are better equipped to assess information about CSAPs and possess a higher likelihood of adoption of CSAPs [61].

The adoption of crop rotation and improved wheat varieties increased with the education level of the household head. Educated wheat producer farmers have more probability to implement crop rotation and improved wheat varieties as compared to uneducated wheat producer farmers. The reason for this could be that increase in awareness and access to information about climate change-related problems, CSAPs, and their benefits are linked to education level, which motivate them to adopt different CSAPs. In addition, educating farmers increases their openness, rationality, and capability to assess the advantages of new technologies. This finding is in line with the findings of [62,63], who reported that the ability of farmers to learn and adopt new ideas is enhanced by education, thereby facilitating the implementation of different CSAPs. Khonje et al. [64] reported that the likelihood of the use of improved maize varieties and conservation agriculture increases with the education level of farmers in Zambia.

Livestock holding had a positive significant association with the adoption of wheat row planting and improved wheat varieties at 1 % and 5 % significant levels, respectively. Livestock play a significant role in agriculture because they provide money to purchase agricultural inputs, including improved seeds and hired labourers during labour shortages because row planting practices are labour-intensive practices. Likewise, the findings of [63,65] reported that the intensity of the implementation of CSAPs increased as ownership of livestock increased. Besides, studies conducted in southern Tanzania by Ref. [66] and western Kenya by Ref. [67] found that the adoption of CSAPs was significantly and positively affected by livestock holding.

Development agent contact positively affected the adoption of wheat row planting, crop rotation, and improved wheat varieties at 1 %, 5 %, and 1 % significant levels, respectively. Many previous studies showed that communication with extension agents was a major factor in the adoption of different CSAPs [42,68,69]. A study conducted by Ref. [62] also revealed that communication between smallholder farmers and development agents is a key conduit for the dissemination of agricultural development research findings. Thus, in turn, promoting the uptake of agricultural technologies. Farmers who frequently communicate with development agent can become more aware of and knowledgeable about issues related to climate change problems, and access information about CSAPs, which facilitate the adoption of CSAPs. A study result conducted by Ref. [20] in Ghana shows that development agents positively influence the adoption of maize row planting. Musafira et al. [67] in western Kenya also found that soil and water conservation practices were positively influenced by development agents.

The adoption of wheat row planting and improved wheat varieties were positively and significantly related to credit access. The possible justification could be that credit is one way of solving financial constraints, which enables smallholder farmers to afford and timely purchase different agricultural inputs and improve labour constraints through hiring labour at times of shortage. Gebremariama and Tesfaye [70] found that credit use significantly and positively affects the adoption of improved agricultural technologies. Similarly [37,71], found that credit utilization helps the household to adopt improved agricultural technologies and practices by improving income constraints that households could face to purchase. Consequently, they apply agricultural inputs on time.

Participation in off-farm activities was negatively and significantly correlated with the adoption of row planting of wheat at a 1 % significant level, while positively and significantly correlated with the use of crop rotation and improved wheat varieties at 1 % and 5 % significant levels, respectively. Row planting of wheat is a labour-intensive CSAP in nature; thus, it is more difficult for farmers to gather enough labour for wheat row planting when they participate in off-farm activities because they compete with agricultural production for labour resources. As a result, farmers who participate in off-farm activities are less likely to adopt row planting of wheat. This finding is in line with the finding of [72], who reported that income obtained from off-farm activities had a negative relation with CSAP adoption intensity in Ghana. Increased off-farm activities are necessary to earn more off-farm earnings, which detracts from labour allocation to farming. The positive relationship between off-farm activities and the adoption of crop rotation and improved wheat varieties is that farmers can generate extra money by engaging in different off-farm activities. This is thought to help them financially so they can buy new agricultural inputs. This finding is in line with the finding of [73], who found that income obtained from off-farm activities increases the probability of the adoption of improved crop varieties, adjust dates of planting, and crop diversification. However, income obtained from off-farm activities negatively affects the adoption of soil and water conservation practices.

Distance to input supply institutions showed a negative and significant relation with the adoption of crop rotation and improved wheat varieties at 1 % and 10 % significant levels, respectively. This could be mainly due to the increment of agricultural input transportation costs as farmers are far away from input supply institutions, which, in turn, increases the cost of production. In addition, proximity to input supply institutions can serve as an inductor of access to information regarding the use and accessibility of agricultural inputs, technology, and practices. Similar findings were also reported in the studies conducted by Refs. [19,74] regarding the relationship between distance to input supply institutions and the adoption of CSAPs in Ethiopia. A study conducted in Vietnam also revealed that market distance negatively affects CSAP adoption decisions [61].

The slope of the plot had negatively affected the wheat row planting adoption decision at a 1 % significance level. If the slope of the plot is flat, then the likelihood of planting of wheat in row decreases. This is because flat slope lands are less vulnerable to soil erosion as compared to steep slope land. This indicates that the likelihood of planting of wheat in row is higher in sloppy lands. Similarly, studies conducted by Refs. [75,76] reported that households with steep-slope farm plots are more inclined to adopt sustainable land management practices than those with flat-slope farm plots. An investigation carried out in Ghana demonstrated that the probability of adoption of row planting increases with steep-slope farm plots [20].

The soil fertility status of the plot is an additional factor influencing the decision to adopt CSAPs. Using fertile plots as a benchmark, soil fertility was positively and significantly associated with the implementation of wheat row planting and crop rotation. The study findings revealed that farmers are more likely to practice crop rotation and planting of wheat in row on medium and less fertile plots than on fertile plots. This result is not surprising; hence, crop rotation is less costly, easier to adopt, and has good potential to improve farm plots’ fertility status. The results of [77] are in line with this investigation. Compared to farmers with fertile plots, those who believed their plots are infertile and moderately fertile have a greater probability to incorporate residues and practice crop rotation. Tekielwold et al. [19] revealed that good soil quality hurts the implementation of CSAPs. Teklu et al. [51] also reported that farmers with poor fertility farmland are more likelihood to use compost than fertile farmland.

## 5 Conclusion and policy implications

This paper aimed to examine the determinants of the adoption of CSAPs in three districts of the east Gojjam zone. This study utilized primary data from 385 wheat producer farmers, encompassing 702 plots, selected through purposive and two-stage sampling methods. The study used a MVP model to identify the factors that influence the adoption decision of CSAPs, such as wheat row planting, crop rotation, and improved wheat varieties. The regression result revealed that the household decision to adopt wheat row planting was significantly and positively affected by livestock holdings, development agents contact, soil fertility status, and credit access, while negatively affected by household head age, off-farm activities participation, and plot slope. Crop rotation was positively influenced by the household head education level, development agents contact, soil fertility status, and off-farm activities participation, while negatively affected by distance to input supply institution. Adoption of improved wheat varieties was positively influenced by the household head education level, livestock holdings, development agents contact, credit access, and off-farm activities participation, while negatively affected by distance to input supply institution.

The results of this study show that the decisions to adopt wheat row planting, crop rotation, and improved wheat variety are not mutually exclusive; rather, the likelihood of adoption was interdependent (complementary). This shows that the adoption of either of the three CSAPs’ exhibited a spillover influence on the implementation of other practices. Thus, a change in policies regarding the implementation of one CSAP would have a spillover effect on the implementation of other CSAPs. Based on these conclusions, we suggest that agricultural development policy should focus on multiple CSAPs. Besides, improving farmers' education level through providing technical and practical training about CSAPs, improving development agents contact, strengthening crop-livestock mixed farming system, and facilitating and strengthening the existing input supply market centers and credit supply institutions are necessary to scale up the adoption of CSAPs.

Although this study is informative, it relies on cross-sectional data, which only records information at a specific moment in time, make it more difficult to determine the causal linkages between the determinants and the adoption of CSAPs. Longitudinal data would be more effective for observing changes and trends over time. This study is limited by its focus on only three CSAPs. This narrow scope may not fully capture the diverse range of CSAPs available and their varying impacts on agricultural productivity and sustainability. The study does not deeply explore the economic aspects of adopting CSAPs, such as cost-benefit analysis, affordability, and financial incentives, which are crucial for understanding the practical feasibility for farmers. By recognizing these limitations, further studies can endeavor to fill in these gaps.

## Data availability statement

Data will be made available on reasonable request.

## CRediT authorship contribution statement

**Sindie Alemayehu:** Writing – review & editing, Writing – original draft, Software, Methodology, Investigation, Formal analysis, Data curation, Conceptualization. **Zemen Ayalew:** Writing – review & editing, Supervision, Methodology, Investigation, Conceptualization, Formal analysis. **Million Sileshi:** Writing – review & editing, Supervision, Methodology, Investigation, Conceptualization, Formal analysis. **Fresenbet Zeleke:** Writing – review & editing, Supervision, Methodology, Investigation, Conceptualization, Formal analysis.

## Declaration of competing interest

The authors declare that they have no known competing financial interests or personal relationships that could have appeared to influence the work reported in this paper.

## Figures and Tables

**Appendix Table 1:** Contingency coefficient among discrete variables

Variables	(1)	(2)	(3)	(4)	(5)	(6)	(7)
Sex	1.000						
Soil fertility status	0.045	1.000					
Off-farm activity	−0.093	−0.058	1.000				
Credit access	0.029	0.004	0.019	1.000			
Cooperative membership	0.160	0.021	−0.075	0.161	1.000		
Training access	−0.038	−0.001	−0.057	0.099	0.037	1.000	
Slope of the plot	−0.001	0.468	−0.003	−0.035	0.025	−0.054	1.000

**Appendix Table 2:** Multicollinearity test for continuous variables

Variables	VIF	1/VIF
Age of the household	1.317	0.759
Family size	1.232	0.812
Education level of the household	1.214	0.824
Livestock holding	1.064	0.94
Distance from input supply institution	1.049	0.954
Number of contact with development agent	1.047	0.955
Plot distance	1.03	0.97
Wheat plot area	1.017	0.983
Mean VIF	1.121	.
