# Supplementary material for: Determinants of the adoption of climate smart agriculture practices by smallholder wheat farmers in northwestern Ethiopia
Source: Heliyon. 2024 Jul 6;10(13):e34233. doi: 10.1016/j.heliyon.2024.e34233 (PMC11292493; doi:10.1016/j.heliyon.2024.e34233)
Supplement: Multimedia component 1 [file mmc1.docx]

**Household Sample Survey Interview Schedule**

**Determinants of Adoption of Climate Smart Agriculture Practices by Smallholder Wheat Farmers in Northwestern Ethiopia**

**PhD Dissertation Research Project**

**Haramaya University**

Household ID (001-385): ___________________

Name of the district: ___________________________________

Kebele: _____________________

Village: _______________

Agro-ecology: 0. Kola 1. Weyina dega 2. Dega

Date of interview: _________________

Name of the enumerator: ___________________________ Signature: ___________

Name of supervisor: ________________________________ Signature: __________

**Section I. Questions on Demographic Characteristics of the Respondents**

1. Name of the household head: ______________________________________________
2. Sex of the household head 1. Male 0. Female
3. Age of household head____________year
4. Marital status of household head

1. Single 2. Married 3. Divorced 4. Widows

1. Education level of the household head ____________ year of formal schooling completed.
2. How many years did you participate in farming activity? _______________
3. Family size of the household

| Family member | Age | Sex (male=1, female=0) | Education level | Job |
| --- | --- | --- | --- | --- |
| 1 |  |  |  |  |
| 2 |  |  |  |  |
| 3 |  |  |  |  |
| 4 |  |  |  |  |
| 5 |  |  |  |  |
| 6 |  |  |  |  |
| 7 |  |  |  |  |
| 8 |  |  |  |  |
| 9 |  |  |  |  |
| 10 |  |  |  |  |
| 11 |  |  |  |  |

**Section II. Questions on Socio-Economic Characteristics of the Respondents**

1. Livestock ownership of the household

| Type | Number owned | Number sold | Unit price | income from sold(birr) |
| --- | --- | --- | --- | --- |
| Cows |  |  |  |  |
| Oxen |  |  |  |  |
| Calves |  |  |  |  |
| Heifer |  |  |  |  |
| Bulls |  |  |  |  |
| Sheep |  |  |  |  |
| Goats |  |  |  |  |
| Donkeys |  |  |  |  |
| Horses |  |  |  |  |
| Mules |  |  |  |  |
| Poultry |  |  |  |  |
| Other |  |  |  |  |
| **Livestock by product sold** | | | | |
| eggs |  |  |  |  |
| Milk |  |  |  |  |
| Butter |  |  |  |  |
| Hide/ Skins |  |  |  |  |

1. Total land holding in 2021/22.

| Types of land | Own land (Timad) | Rented in land (Timad) | Rented out land (Timad) | Shared land |
| --- | --- | --- | --- | --- |
| Cultivated land |  |  |  |  |
| Grazing land |  |  |  |  |
| Forest land |  |  |  |  |
| Others |  |  |  |  |

1. Did you have land certificate? 1. Yes 0. No
2. How many crops did you produce within the 2021/22 cropping year? ____________
3. Please tell me your land holding size and the crops grown within the 2021/22 cropping year.

| Type of crop grown | Area allocated (timad) | Quantity produced(quintal) | Quantity sold (quintal) | Stock | Unit price |
| --- | --- | --- | --- | --- | --- |
| Teff |  |  |  |  |  |
| Wheat |  |  |  |  |  |
| Maize |  |  |  |  |  |
| Sorghum |  |  |  |  |  |
| Barley |  |  |  |  |  |
| Nuge |  |  |  |  |  |
| Millet |  |  |  |  |  |
| Chickpea |  |  |  |  |  |
| Bean |  |  |  |  |  |
| Potato |  |  |  |  |  |
| Other |  |  |  |  |  |
| Revenue from crop residue/ straws | | |  |  |  |

1. How much income did you get from the sales of vegetables and fruits in the past 12 months in Birr? ____________ sale of eucalyptus in the past 12 months in Birr? ____________sale of chat in the past 12 months in Birr? ____________
2. How many wheat farm plots do you have in 2021/22 cropping years? ____________
3. Please tell me your wheat farm plot level characteristics?

| No | Description (please specify your answer for each wheat farm plot) | Plot 1 | Plot 2 | Plot 3 | Plot 4 |
| --- | --- | --- | --- | --- | --- |
| 1 | Wheat Plot ownership 1=owned 2= rented in 3=Rented out 4=Shared |  |  |  |  |
| 2 | The area of each wheat plot (in timad) |  |  |  |  |
| 3 | How is the slope of your plots? 1. Steep slope, 0. Flat slope |  |  |  |  |
| 4 | How much time it takes from home to your plot in walking minutes? |  |  |  |  |
| 5 | Do you think that your plot is highly susceptible to soil erosion?  1. Yes 0. No |  |  |  |  |
| 6 | What is the soil fertility status of your plot?  1. Highly fertile 2. Medium 3. Low |  |  |  |  |
| 7 | Did you use climate smart agriculture practices own your wheat farm plots? 1. Yes 0. No |  |  |  |  |
| 8 | Which climate smart agriculture practices do you use in wheat growing plot? Please write ‘1’ for Yes and ‘0’ for No to each wheat farm plot. |  |  |  |  |
| 8.1 | Reduced tillage 1. Yes 0. No |  |  |  |  |
| 8.2 | Crop residue management 1. Yes 0. No |  |  |  |  |
| 8.3 | Crop rotation 1. Yes 0. No |  |  |  |  |
| 8.4 | Compost and manure used 1. Yes 0. No |  |  |  |  |
| 8.5 | Used improved wheat varieties 1. Yes 0. No |  |  |  |  |
| 8.6 | Row planting 1. Yes 0. No |  |  |  |  |
| 8.7 | Early planting 1. Yes 0. No |  |  |  |  |
| 8.8 | Small-scale irrigation 1. Yes 0. No |  |  |  |  |
| 8.9 | Tree-based conservation agriculture 1. Yes 0. No |  |  |  |  |

1. **Information on production cost of wheat**

| Input Costs | | | Unit |  | | Total quantity | | | Unit Price | Total Cost |
| --- | --- | --- | --- | --- | --- | --- | --- | --- | --- | --- |
|  |  |  |  | P-1 | P-2 | | p-3 | P-4 |  |  |
| Seed | Local | | Kg |  |  | |  |  |  |  |
|  | improved | | Kg |  |  | |  |  |  |  |
| Fertilizer | DAP | | Kg |  |  | |  |  |  |  |
|  | UREA | | Kg |  |  | |  |  |  |  |
| Organic Fertilizer | Manure | | Quintal |  |  | |  |  |  |  |
|  | Compost | | Quintal |  |  | |  |  |  |  |
| Land | | | Ha |  |  | |  |  |  |  |
| Chemicals | | Fungicide | Li |  |  | |  |  |  |  |
|  |  | Herbicide | Li |  |  | |  |  |  |  |
|  |  | Pesticide | Li |  |  | |  |  |  |  |

1. Did you use irrigation water to produce crops? 1. Yes 0. No
2. If your answer is yes for Q-17, what proportion of your cropland was irrigated? ______(timad)
3. Do you or your family members participate in off-farming activities? 1. Yes 0. No
4. If your answer is yes, Specify the type of activity you or your family engaged?

| Type of activity household member participate | Annual income obtained |
| --- | --- |
| Petty trading |  |
| Handicrafts |  |
| Sale fuel wood and charcoal |  |
| Daily laborer |  |
| Employed |  |
| Renting Asset |  |
| Other |  |

**Section III. Questions on institutional factors**

1. Distance of your residence from the nearest market center in walking minutes _______
2. Distance of your residence from the nearest input supply market (institution) in walking minutes____________
3. Distance of your residence from the development agent office in walking minutes _______
4. Distance of your residence from the training center in walking minutes _______
5. Distance of your residence from the cooperatives office in walking minutes _______
6. Do you have contact with development agent in the 2021/22 cropping season? 1. Yes 0. No
7. If yes, how many times do you contact with development agent? -----------day/year
8. In what season more frequently contact with them?

1. Sowing season 2. Harvesting time 3. Throughout the year

1. Do you take credit in 2021/22? 1. Yes 0. No
2. If yes, how much do you take? --------------Birr.
3. From whom do you get credit?

1. Micro finance institution 2. Cooperatives 3. Bank 4. Traders 5. Friends 6. other______

1. For what purpose did you take the credit?

1. Fertilizer 2. To purchase farm equipment 3. To rent in land 4. For Seed 5. Other______

1. Do you are members of agricultural cooperatives? 1. Yes 0. No
2. Did you get training related to climate smart agriculture practices? 1. Yes 0. No

**Section IV. Climate change and variability questions**

1. Did you get information about climate change and weather condition? 1. Yes 0. No
2. If your answer is yes for Q-34, from home you get the information?

1.Development agents 2. Cooperatives 3. Metrological experts

4. Radio/Television 5. Other ___________

1. What is your observation/perception on climate variability, soil fertility status and crop production around your environment in this year than: (Using the following Key: 0 no change, 1 very low, 2 low, 4 high and 5 Very high put (√) in the box provided?

| Indicator | 30 years ago | 10 years ago | 2 years ago |
| --- | --- | --- | --- |
| Temperature |  |  |  |
| Rainfall |  |  |  |
| Soil fertility status |  |  |  |
| Crop production |  |  |  |

1. Do you get information about climate smart agriculture practices? 1. Yes 0. No
2. If your answer is yes for Q-37, from home you get the information?

1**.** Development agents 2. Cooperatives 3. Metrological experts

4. Radio/Television 5. Other ___________
